# Hydrogels Embedded With Melittin and Tobramycin Are Effective Against *Pseudomonas aeruginosa* Biofilms in an Animal Wound Model

**DOI:** 10.3389/fmicb.2019.01348

**Published:** 2019-06-20

**Authors:** Michael M. Maiden, Mitchell P. Zachos, Christopher M. Waters

**Affiliations:** ^1^Department of Microbiology and Molecular Genetics, Michigan State University, East Lansing, MI, United States; ^2^The BEACON Center for the Study of Evolution in Action, Michigan State University, East Lansing, MI, United States

**Keywords:** hydrogels, IVIS, *Pseudomonas aeruginosa*, *Staphylococcus aureus*, biofilms, antimicrobial peptides

## Abstract

We demonstrate that the antimicrobial peptide, melittin, is effective alone and in combination with the aminoglycosides tobramycin to kill *Pseudomonas aeruginosa* growing as biofilms both *in vitro* and *in vivo*. Melittin and tobramycin show enhanced *in vitro* activity in combination at micromolar concentrations, resulting in a 2-log_10_ reduction in the number of cells within mature PAO1 *P. aeruginosa* biofilms after 6-h of treatment. Alternatively, either agent alone resulted in half-a-log_10_ reduction. Time-killing assays demonstrated that the combination of melittin and tobramycin was effective at 2-h whereas tobramycin was not effective until after 6-h of treatment. We also found the combination was more effective than tobramycin alone against biofilms of 7 *P. aeruginosa* cystic fibrosis clinical isolates, resulting in a maximum 1.5-log_10_ cellular reduction. Additionally, melittin alone was effective at killing biofilms of 4 *Staphylococcus aureus* isolates, resulting in a maximum 2-log_10_ cellular reduction. Finally, melittin in combination with tobramycin embedded in an agarose-based hydrogel resulted in a 4-fold reduction in bioluminescent *P. aeruginosa* colonizing mouse wounds by 4-h. In contrast, tobramycin or melittin treatment alone did not cause a statistically significant reduction in bioluminescence. These data demonstrate that melittin in combination with tobramycin embedded in a hydrogel is a potential treatment for biofilm-associated wound infections.

## Introduction

Biofilms represent a unique challenge in that they possess several antibacterial tolerance mechanisms, contributing to resistance and negating our antibacterial arsenal (Lewis, 2001; Brown and Wright, 2016). Biofilms consist of cells imbedded in an extracellular polymeric substance made up of polysaccharides, proteins, and DNA that creates a barrier to the host immune system, slows the diffusion of antimicrobials and gives rise to slow-growing or dormant persister cells (Donlan and Costerton, 2002). These tolerance mechanisms render cells growing as biofilms up to 1,000-times more resistant to antibacterial therapies compared to planktonic cells (Lewis, 2001).

Members of the multidrug-resistant “ESKAPE” pathogens (*Enterococcus faecium, Staphylococcus aureus, Klebsiella pneumoniae, Acinetobacter baumannii, Pseudomonas aeruginosa*, and *Enterobacter species)* form biofilms in non-healing diabetic ulcers, burn wounds and the lungs of patients with cystic fibrosis (CF) (Altoparlak et al., 2004; Sivanmaliappan and Sevanan, 2011; Omar et al., 2017). Few antimicrobial therapies are effective against ESKAPE pathogens, especially in a biofilm state, and they are a major cause of morbidity and mortality in CF and infection in chronic non-healing wounds in diabetic patients (Emerson et al., 2002; Heijerman, 2005; Frykberg and Banks, 2015). As virtually all antimicrobials were developed against planktonically growing bacteria, there is a vital need for new antimicrobials that more effectively target bacteria growing as recalcitrant biofilms.

Antimicrobial peptides (AMPs) represent a promising therapeutic option for biofilm-associated infections. AMPs, or “natures antibiotics,” are found in most branches of life and offer key advantages over traditional antimicrobials in that they rapidly kill and are effective against slow-growing or dormant bacteria (Papagianni, 2003; Hancock and Sahl, 2006). Importantly, AMPs have a high affinity for bacterial membranes. First, the positive charge found in all AMPs is highly selective for the negative anionic phospholipids that make up bacterial membranes (Yeaman and Yount, 2003). Second, AMPs specificity for bacteria is driven by their highly negatively charged interiors, which further drives AMP binding (Yeaman and Yount, 2003).

The AMP melittin is derived from the venom of European honey bee *Apis mellifera* and is a cationic amphiphilic linear peptide (NH_2_-GIGAVLKVLTTGLPALISWIKRKRQQ-CONH2) that causes membrane permeabilization (Raghuraman and Chattopadhyay, 2006). At high concentrations, melittin can induce pain and inflammation in humans; however, concentrations up to ∼35 mM have been shown to have anti-nociceptive and anti-inflammatory properties in animal models (Raghuraman and Chattopadhyay, 2006; Lee et al., 2014; Lee and Bae, 2016). Melittin has also been shown to have antibacterial properties against *S. aureus* and *P. aeruginosa*, both inhibiting biofilm formation and causing biofilm dispersal (Dosler and Karaaslan, 2014; Choi et al., 2015; Lee and Bae, 2016; Picoli et al., 2017). Melittin has also been tested alone and in combination with several antibiotics against *Acinetobacter baumannii* and polymicrobial environmental biofilms isolated from the dairy industry (Bardbari et al., 2018; Galdiero et al., 2019; Pashaei et al., 2019).

Here, we evaluated melittin for activity in combination with aminoglycosides against mature biofilms formed by *P. aeruginosa*. We found that the combination of tobramycin with melittin showed non-additive enhanced activity *in vitro* to eradicate biofilms of multiple clinical isolates of *P*. *aeruginosa*. Furthermore, this combination was effective at killing biofilms of *Staphylococcus aureus in vitro*, primarily due to melittin activity. Finally, we developed a novel hydrogel formulation embedded with melittin and/or tobramycin and only observed significant killing of *P. aeruginosa* biofilms in a murine wound model after 4-h of treatment with the combination. Our findings suggest that melittin in combination with tobramycin could represent a potential new therapy for the treatment of biofilm-associated infections in diabetic foot and burn wounds as applied to the surface in a hydrogel.

## Materials and Methods

### Bacterial Strains, Culture Conditions, and Compounds

All strains used in this study are listed in Table 1. Bacterial strains were grown in glass test tubes (18 × 150 mm) at 35°C in cation adjusted Müeller-Hinton Broth II (MHB II, Sigma-Aldrich) with agitation at 210 revolutions per minute (RPM). Antibiotics and melittin were purchased from Sigma-Aldrich. Melittin used in this study was ≥85% pure and was derived from honey bee venom. Melittin can induce an IgE response in 1/3rd of patients sensitive to honeybee venom; however, it has been speculated this is due to additional compounds found in bee venom (Lee and Bae, 2016). Synthetically produced melittin is potentially more efficacious, less allergenic and will be evaluated in future studies. Tobramycin sulfate, gentamicin sulfate, and streptomycin sulfate were dissolved in autoclaved deionized water and filter sterilized using 0.22 μM filter membranes (Thomas Scientific). A stock solution of 1 mM of melittin was dissolved in dimethyl sulfoxide (DMSO). In a control experiment we determined that at the concentrations used DMSO has minimal effects on mature *P. aeruginosa* biofilms (Supplementary Figure S1).

**Table 1:** Bacterial strains used in this study.

Strain	General characteristics and known resistance and susceptibilities		References
PAO1	PA standard reference strain, isolated in 1954 (Holloway, 1955)	tob^s^, gent^s^, strep^s^	Maiden et al., 2018a
Xen41	Bioluminescent PAO1 derivative: constitutively expresses *luxCDABE* gene	tob^s^, gent^s^, strep^s^	PerkinElmer
CF_110_N^+^	Michigan PA clinical CF isolate, from patient 110	tob^r^	Maiden et al., 2018a
CF_110_O^+^	Michigan PA clinical CF isolate, from patient 110	tob^s^	Maiden et al., 2018a
CF_115_J	Michigan PA clinical CF isolate, from patient 115	tob^s^	Maiden et al., 2018a
CF_131_M	Michigan PA clinical CF isolate, from patient 131	tob^s^	Maiden et al., 2018a
CF_300_A	Michigan PA clinical CF isolate, from patient 300	tob^r^	Maiden et al., 2018a
AMT0023_30^∗^	PA early isolate 6 MO	tob^s^	De Soyza et al., 2013
AMT0023_34^∗^	PA late isolate 8 YO MexXY efflux pump mutant	tob^r^	De Soyza et al., 2013
USA_300_JE2	MRSA, wound isolate, California	tob^r^	Fey et al., 2012
COL	MRSA, Colindale Hospital isolate, England	tob^r^	de Lencastre and Tomasz, 1994
Newman (25904)	MSSA, endocarditis isolate, ATCC	tob^r^	Baba et al., 2007
Wichita (29213)	MSSA, better biofilm former, ATCC	tob^r^	Soni et al., 2015

*PA, Pseudomonas aeruginosa; MRSA, Methicillin-resistant Staphylococcus aureus; MSSA, Methicillin-sensitive Staphylococcus aureus; ATTC, American Tissue Type Collection; YO, years old; MO, month; tob, tobramycin; gent, gentamicin; strep, streptomycin. ^∗^Taken from same patient at 6 MO and 8 YO. ^+^Isolates were taken from same patient 3 MO apart.*

### Minimum Inhibitory Concentration (MIC)

Minimum inhibitory concentrations were determined as described previously (Andrews, 2001). Briefly, microdilutions of PAO1 *P. aeruginosa* were made in a 96-well plate in 10% (v/v) MHB II diluted in Dulbecco’s Phosphate Buffered Saline with magnesium and calcium (DPBS, Sigma-Aldrich). ∼1 × 10^6^ colony forming units/mL (CFUs/mL) were added and incubated for 24-h at 35°C with agitation at 150 RPM. MICs were chosen as the minimum concentration in which no turbidity greater than background was measured (absorbance at 595 nm) using a SpectraMax M5 microplate spectrophotometer system (Molecular Devices).

### Biofilm Susceptibility Testing Using BacTiter-Glo^TM^

To measure biofilm antimicrobial susceptibility against the strains of *P. aeruginosa* listed in Table 1, the MBEC^TM^ assay was used (Innovotech) as previously described (Maiden et al., 2018a). Briefly, an overnight culture was washed and diluted to an OD_600_ of 0.001 and seeded into a MBEC^TM^ plate and incubated for 24-h at 35°C with agitation at 150 RPM. After 24-h, the lid was then washed for 5-min to remove non-adherent cells. The lid was transferred to a 96-well treatment plate and incubated for the indicated time at 35°C without agitation. Following treatment, the MBEC^TM^ lid was washed and transferred to a black 96-well ViewPlate (PerkinElmer) filled with 40% (v/v) BacTiter-Glo^TM^ (Promega) diluted in DPBS to enumerate cell viability using luminescence by an EnVison Multilabel Plate Reader (PerkinElmer). The BacTiter-Glo^TM^ microbial cell viability assay is a luminescent assay that determines the number of viable cells present based on quantification of adenosine triphosphate concentration. A calibration curve was previously performed, and it was found to be *r*^2^ = 0.9884 for luminescence versus CFUs/mL, indicating BacTiter-Glo^TM^ is an effective measurement of cell viability (Maiden et al., 2018a). Dose response curves (DRCs), checkerboard assays, and time killing curves were performed similarly. To test for antimicrobial susceptibility against the strains of *S. aureus* listed in Table 1, biofilms were formed using a standard 96-well ViewPlate as we found *S. aureus* did not form biofilms on MBEC^TM^ pegs at the air-liquid interface.

### Crystal Violet Staining

To study biofilm dispersal under static conditions, crystal violet staining was performed as previously described (Sambanthamoorthy et al., 2011). Briefly, biofilms were formed by PAO1 *P. aeruginosa* on MBEC^TM^ plates as described above and then stained with crystal violet following 6-h treatments.

### Membrane Permeabilization Assay

24-h old biofilms were formed by POA1 *P. aeruginosa* in glass test tubes (18 × 150 mm) in 1 mL of 10% (v/v) MHB II at 35°C and agitated at 150 RPM as previously described (Maiden et al., 2018b). Cells were then washed in DPBS to remove non-adherent cells and treated with melittin and tobramycin for 2-h. Following treatment cells were washed in PBS (phosphate buffered solution without magnesium and calcium) and the biofilm was disrupted from the air-liquid interface using an autoclaved wooden stick. The cells were stained with TO-PRO-3 iodide, which fluoresces in cells that have compromised membranes by intercalating DNA. Single cell flow cytometry was performed on an LSR II (BD Biosciences) with excitation from the 640 mm laser.

### Agarose Hydrogels

Agarose hydrogels were made by dissolving 1 gm of agarose (Sigma-Aldrich) into 200 mL of Tris-acetate-EDTA buffer and heated to form a homogenous solution using a microwave. The 0.5% agarose solution was then allowed to cool and various treatments were added. The solution was then poured into 100 × 15 mm petri dishes (Thermo Fisher Scientific) and stored at 4°C overnight. Prior to treatment, a 4 mm biopsy punch (VWR) was used to create hydrogel wafers.

### Murine Wound Infection Model

Wound surgery was performed on 8–9-week-old male and female SKH-1 mice (Charles River) as previously described (Agostinho Hunt et al., 2017; Hunt et al., 2017). 24-h old wounds were infected with ∼1 × 10^9^ Xen41 *P. aeruginosa* cells (PerkinElmer), which is a bioluminescent derivative of PAO1 that constitutively expresses the *luxCDABE* genes. Briefly, 24-h old biofilms were formed on sterilized polycarbonate membrane filters with a 0.2 μM pore size (Millipore Sigma) by diluting an overnight culture to an OD_600_ of 0.001 and pipetting 100 μl on 4 membranes on a tryptic soy agar (TSA) plate. 24-h old biofilms were scraped using L-shaped spreaders (Sigma-Aldrich) from each membrane and re-suspended in 500 μl of DPBS. 20 μl of the biofilm-suspensions were inoculated into 24-h old wounds formed on the dorsal side of the mouse midway between the head and the base of the tail. 24-h later the biofilm was imaged using the *In Vivo* Imaging System (IVIS, Perkin Elmer). The biofilm was then treated by placing a 4 mm 0.5% agarose hydrogel on the wound for 4-h. The biofilm was imaged before and after treatment and total flux (photons/sec) was used to quantify bacterial susceptibility. Representative IVIS images are shown in Supplementary Figure S2.

### Ethics Statement

This study was carried out in accordance with the Michigan State University Institutional Animal Care and Use Committee (application 03/18-036-00). MSU is accredited by the Association for Assessment and Accreditation of Laboratory Animal Care (AAALAC) and ensures compliance with federal regulatory requirements.

## Results

### Melittin Alone and in Combination With Tobramycin or Gentamicin Is More Effective at Killing PAO1 *P. aeruginosa* Biofilms Than Each Aminoglycoside Alone

**FIGURE 1:**
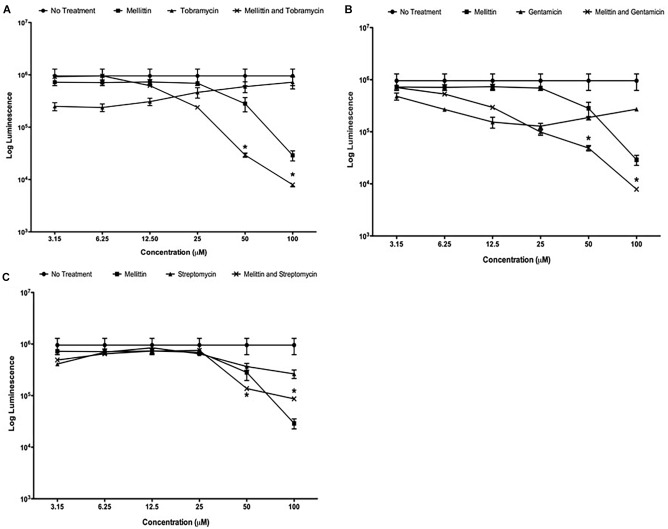
Melittin alone and in combination with tobramycin, gentamicin or streptomycin is more effective than each aminoglycoside alone. PAO1 *P. aeruginosa* biofilms were treated using two-fold dilutions of melittin alone and in combination with **(A)** tobramycin, **(B)** gentamicin and **(C)** streptomycin. The number of viable cells was quantified using BacTiter-Glo^TM^. Each assay was performed twice in triplicate. The results represent means ± Standard Error Mean (SEM). Melittin and no treatment were re-plotted in each panel for comparison. A two-way analysis of variance (ANOVA) was performed followed by a Tukey’s post-tests to determine statistical significance between each aminoglycoside and the combination (^∗^*p* < 0.05).

We previously demonstrated that the compounds triclosan and oxyclozanide synergize with tobramycin to reduce both Gram-negative and Gram-positive biofilms (Maiden et al., 2018a,b). During these studies, we used melittin as a positive control for cell permeabilization and observed that it had potent activity against *P. aeruginosa* biofilms. To further explore this finding, DRCs of melittin were performed alone and in combination with tobramycin, gentamicin or streptomycin against mature *P. aeruginosa* biofilms. Melittin was most effective at 100 μM, resulting in a ∼1.5-log_10_ reduction in the number of cells within a biofilm compared to untreated controls after 6-h of treatment (Figure 1A). Tobramycin alone showed a maximal activity of ∼half-a-log_10_ of cells within biofilms although this effect was observed at lower concentrations of tobramycin. However, when used together, enhancement was seen when 50 μM of melittin was combined with 50 μM of tobramycin, resulting in ∼1.5-log_10_ cellular reduction compared to untreated controls, and 100 μM of melittin and tobramycin produced a ∼2-log_10_ cellular reduction that was significantly more effective than melittin alone. DRCs were also performed with gentamicin or streptomycin combined with melittin (Figure 1B,C). Melittin alone and no treatment were re-plotted in each panel for comparison. The combination of gentamicin and melittin was significantly more effective than either treatment at 50 and 100 μM; however, streptomycin showed little enhancement of melittin at any concentration. This is not surprising as it is known that streptomycin has reduced activity against *P. aeruginosa* and is not used clinically (Gilbert, 2013). These data indicate that melittin alone is effective at reducing biofilms and has greater efficacy (defined as maximum cellular reduction at a given concentration) in combination with tobramycin or gentamicin.

### Melittin Is More Potent in Combination With Tobramycin or Gentamicin

Effective concentration 50 (EC_50_) values were calculated to determine the potency of the combinations against *P. aeruginosa* PAO1 biofilms. The EC_50_ value for melittin was decreased 2.8 and 4.6-fold when used in combination with tobramycin or gentamicin, from 46 to 16 (14–19 μM) and to 10.68 μM (9–11 μM), respectively (95% confidence intervals) (Table 2). An EC_50_ value was ambiguous for the streptomycin and melittin combination because streptomycin failed to significantly potentiate melittin. EC_50_ values for tobramycin, gentamicin or streptomycin when used alone could not be determined using the concentrations tested against cells growing as biofilms as they demonstrated decreased efficacy at higher concentrations. This could be due to the previously described paradoxical effects of aminoglycosides in which higher concentrations are less effective than lower concentrations (Lorian et al., 1979; Gilleland et al., 1989; Barclay and Begg, 2001).

### Melittin Activity Is Only Enhanced by Tobramycin or Gentamicin Against Biofilm-Growing Bacteria

Minimum inhibitory concentrations on PAO1 were performed to determine if melittin in combination with tobramycin, gentamicin, or streptomycin were more effective against planktonic cells. For planktonic cells, all aminoglycosides demonstrated lower MICs than melittin. In addition, the MIC values for each aminoglycoside did not change when used in combination with melittin, suggesting enhancement only occurs against PAO1 *P. aeruginosa* growing as biofilms (Supplementary Table S1).

### Melittin Alone and in Combination With Tobramycin Has a Shorter Onset of Action

**FIGURE 2:**
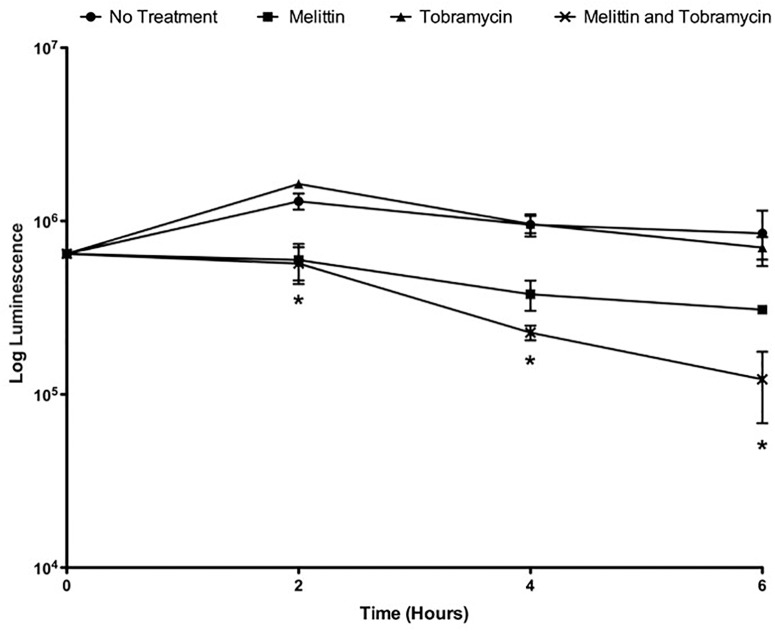
Melittin alone and in combination with tobramycin has a shorter onset of action. PAO1 *P. aeruginosa* biofilms were treated with melittin (50 μM) and tobramycin (400 μM). At 0, 2, 4, and 6-h the number of viable cells within the biofilms were determined by BacTiter-Glo^TM^. Each assay was performed twice in triplicate. The results represent means ± SEM. A two-way ANOVA was performed followed by a Tukey’s post-tests to determine statistical significance between tobramycin alone and the combination (^∗^*p* < 0.05).

Because gentamicin and tobramycin when combined with melittin exhibited similar enhancement of biofilm killing of *P. aeruginosa* PAO1, for the remainder of this work we studied the activity of the combination of tobramycin and melittin. Time-killing curves were performed to study the pharmacokinetic properties of melittin alone and in combination with tobramycin. To perform time-killing curves, the number of viable cells within the biofilms were determined by BacTiter-Glo^TM^ at 0, 2, 4, and 6-h. 50 μM melittin alone or in combination with 400 μM of tobramycin showed activity by 2-h, whereas tobramycin treatment alone was not effective at 6-h (Figure 2). By 6-h, the combination resulted in ∼1-log_10_ cellular reduction whereas melittin alone resulted in ∼half-a-log_10_ cellular reduction and tobramycin exhibited no significant killing. It is not surprising that tobramycin was ineffective as it is known to penetrate the biofilm poorly and can require up to 24-h to diffuse into biofilms (Tseng et al., 2013).

**Table 2:** EC_50_ values for aminoglycoside combinations.

Antibiotic	Antimicrobial peptide	EC_50_ (μM)	95% Confidence Interval (μM)
Melittin	–	46	39–60
Tobramycin	Melittin	16	14–19
Gentamicin	Melittin	10	9–11
Streptomycin	Melittin	N/A	N/A

*EC_50_ values could not be calculated for aminoglycosides when used alone over the concentrations tested against P. aeruginosa growing as a biofilm. EC_50_, half effective concentration 50; N/A, not applicable.*

**FIGURE 3:**
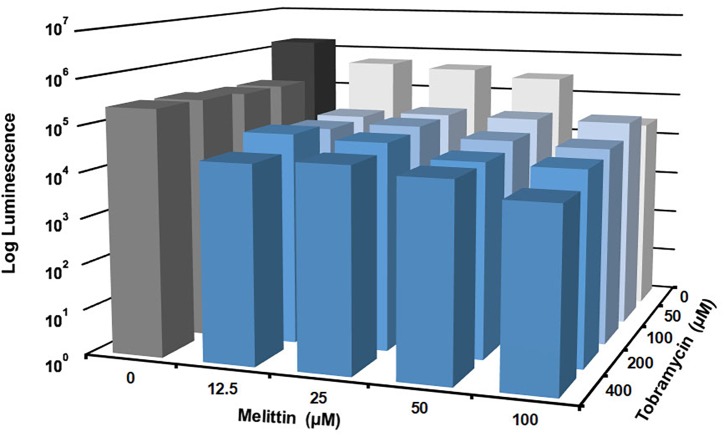
Melittin in combination with tobramycin is additive and synergistic. PAO1 *P. aeruginosa* biofilms were treated for 6-h with checkerboard dilutions of melittin combined with tobramycin. Number of viable cells within the biofilms were quantified by BacTiter-Glo^TM^. The assay was performed twice in triplicate. The results represent means.

**FIGURE 4:**
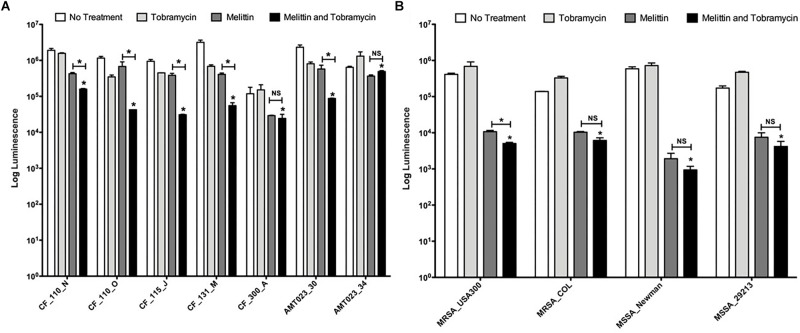
Melittin alone and in combination with tobramycin is effective against *P. aeruginosa and S. aureus* isolates. **(A)** Biofilms formed by CF *P. aeruginosa* isolates were treated with melittin (50 μM) or tobramycin (400 μM) alone and in combination. The number of viable cells was quantified using BacTiter-Glo^TM^. The assay was performed twice in triplicate. The results represent means plus SEM. A one-way ANOVA was performed for each CF clinical isolate followed by a Bonferroni’s post-tests to determine statistical significance between tobramycin and the combination and between melittin and the combination as indicated by black bars (^∗^*p* < 0.05). **(B)** Biofilms formed by *S. aureus* isolates were treated with melittin (100 μM) or tobramycin (100 μM) alone and in combination. The number of viable cells was quantified using BacTiter-Glo^TM^. The assay was performed twice in triplicate. A two-way ANOVA was performed followed by a Tukey’s post-tests to determine statistical significance between tobramycin and the combination and between melittin and the combination as indicated by black bars (^∗^*p* < 0.05). NS, not significant.

### Melittin in Combination With Tobramycin Exhibits Non-additive Enhancement of *P. aeruginosa* Biofilm Killing

Checkerboard experiments were performed to determine the concentrations of melittin and tobramycin that was effective against *P. aeruginosa* biofilms. Melittin was effective alone at 100 μM, resulting in a ∼1.5-log_10_ cellular reduction compared to untreated controls (Figure 3). The maximal effect was observed when 100 μM of melittin was combined with 400 μM of tobramycin, resulting in ∼2-log_10_ cellular reduction compared to untreated controls. Melittin and tobramycin showed enhancement when used in combination between 12.5 and 50 μM of melittin and between 50 and 400 μM of tobramycin, resulting in a ∼1-log_10_ cellular reduction within biofilms compared to either tobramycin or melittin alone (statistical significance is shown in Supplementary Table S2).

### Melittin Alone and in Combination With Tobramycin Is Effective Against *P. aeruginosa* and *S. aureus* Clinical Isolates

We tested the efficacy of tobramycin, melittin, and the combination to kill biofilm-growing bacteria from 7 *P. aeruginosa* CF clinical isolates and 4 *S. aureus* clinical isolates (strains are described Table 1). CF_110_N and CF_110_O were isolated longitudinally from the same patient 3 months apart and AMT0023_30 and 34 were isolated longitudinally from the same patient at 6 months and 8 years of age, respectively (De Soyza et al., 2013; Maiden et al., 2018a).

Melittin (50 μM) in combination with tobramycin (400 μM) significantly killed 7/7 *P. aeruginosa* CF isolates grown as biofilms compared to tobramycin treatment alone, resulting in a maximal ∼1.5-log_10_ cellular reduction compared to untreated controls (CF_115_J) (Figure 4A). Importantly, the combination significantly enhanced killing of strain AMT0023_34 compared to tobramycin alone, which over expresses the RND-type MexXY-OpRM efflux pump, rendering it resistant to tobramycin (Mulcahy et al., 2010). In 5/7 isolates, the combination was significantly more effective than melittin alone, but this was not the case for strains CF_300_A and AMT023_34, suggesting killings of these biofilms is primarily driven by melittin.

Melittin (100 μM) alone was also effective at killing biofilms of 4/4 *S. aureus* isolates tested, resulting in a maximal ∼3-log_10_ cellular reduction compared to controls (MSSA_Newman) (Figure 4B). The limit of detection in this assay is 10^3^. Interestingly, tobramycin (100 μM) was ineffective against each strain of *S. aureus* tested and resistance was observed, but the combination remained effective at killing *S. aureus* biofilms. For 3/4 of the *S. aureus* strains, the combination of tobramycin and melittin was similar to melittin alone, while a slight but significant enhancement was observed for MRSA_USA300. These results suggest that melittin and not tobramycin primarily drives killing of *S. aureus* biofilms.

**FIGURE 5:**
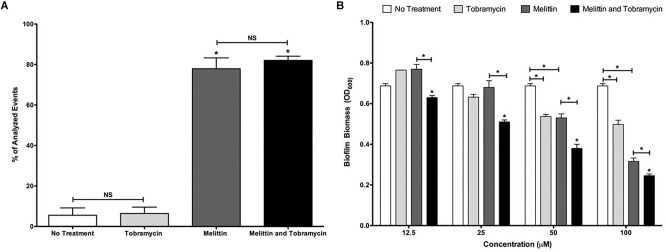
Melittin alone and in combination with tobramycin causes cellular permeabilization and the dispersal of biofilms formed by PAO1 *P. aeruginosa*. **(A)** Biofilms were treated with melittin (100 μM), or tobramycin (400 μM), alone and in combination for 2-h. Cells were stained with TO-PRO-3 to determine the number of cells that were permeabilized. The experiment was performed two separate times in duplicate. The results are percent averages plus the SEM. A one-way ANOVA followed by Bonferroni’s post-tests was used to determine statistical significance between tobramycin and melittin or the combination and as indicated by black bars (^∗^*p* < 0.05). **(B)** Biofilms were treated for 6-h and biofilm dispersal was quantified using crystal violet staining. The assay was performed at least three times in triplicate. The results represent means plus SEM. A two-way ANOVA was performed followed by a Tukey’s post-tests to determine statistical significance between tobramycin and the combination and as indicated by black bars (^∗^*p* < 0.05). NS, not significant.

### Melittin Alone and in Combination With Tobramycin Causes Permeabilization and Biofilm Dispersal

The mechanism of action of AMPs against cells within biofilms is likely through cellular permeabilization (Melo et al., 2009). To test this hypothesis, we used the TO-PRO-3 dye which stains DNA in permeabilized cells with compromised membranes and analyzed treated *P. aeruginosa* biofilms by single cell analysis using flow cytometry. Tobramycin (400 μM) treatment alone did not increase the population of permeabilized cells compared to no treatment, but after 2-h of treatment, 100 μM of melittin significantly increased the population of permeabilized cells within biofilms to 78% compared to 6% for untreated controls (Figure 5A). A similar increase was observed for the combination treatment and this was not significantly different than melittin alone.

We also hypothesized that melittin in combination with tobramycin may cause increased biofilm dispersal. To test this hypothesis, we measured biofilm dispersal by staining the biofilm biomass after treatment using crystal violet. At all concentrations tested the combination resulted in significant biofilm dispersal compared to either melittin or tobramycin treatment alone, and 50 or 100 μM of melittin or tobramycin alone resulted in significant biofilm dispersal compared to untreated controls (Figure 5B). These data suggest that the mechanism of action of melittin alone and in combination with tobramycin may be biofilm dispersal and cellular permeabilization.

**FIGURE 6:**
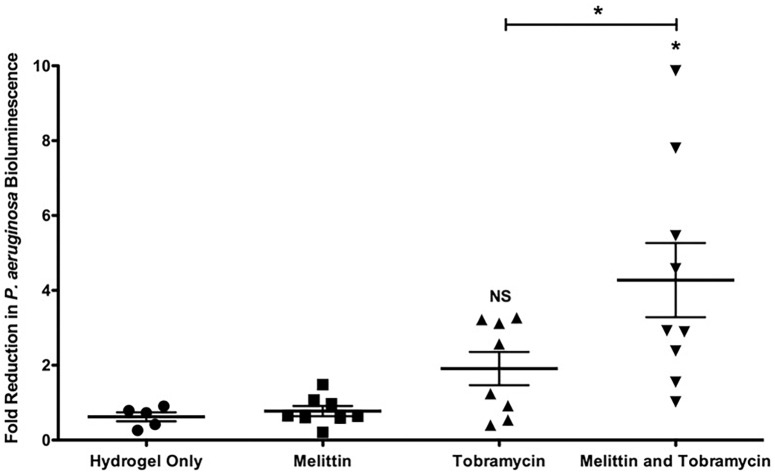
Melittin in combination with tobramycin in a hydrogel is effective *in vivo* against Xen41 *P. aeruginosa*. 24-h old bioluminescent biofilms formed within wounds were treated with melittin (100 μM), or tobramycin (400 μM), alone and in combination for 4-h. Reduction in the number of cells within biofilms was quantified using IVIS. The results are fold reduction of two separate experiments ± SEM, control hydrogels *n* = 5, melittin hydrogels *n* = 8, tobramycin hydrogels *n* = 8, melittin and tobramycin hydrogels *n* = 9. A one-way ANOVA followed by Bonferroni’s post-tests was used to determine statistical significance between each treatment and controls and as indicated by black bars (^∗^*p* < 0.05). NS, not significant.

### Melittin in Combination With Tobramycin Imbedded in an Agarose-Based Hydrogel Is Effective Against *P. aeruginosa* Biofilms in a Murine Wound Model

To determine if melittin and tobramycin were effective against Xen41 *P. aeruginosa* biofilms *in vivo*, we tested their activity using an IVIS murine wound model (Agostinho Hunt et al., 2017; Hunt et al., 2017). Xen41 *P. aeruginosa* is a bioluminescent derivative of PAO1 that constitutively expresses the *luxCDABE* genes. Mature biofilms colonizing an open wound were treated for 4-h using a hydrogel imbedded with either 100 μM of melittin or 400 μM of tobramycin alone and in combination. This combination was chosen because it caused the maximum amount of *in vitro* killing (Figure 3). Hydrogels containing melittin and tobramycin resulted in a significant 4.2-fold-reduction in biofilm bioluminescence compared to tobramycin treatment alone or animals treated with the control hydrogel (Figure 6). The combination also resulted in a maximal reduction in biofilm bioluminescence of 7.8 and 9.8-fold. Hydrogels containing only tobramycin resulted in 1.8-fold-reduction in biofilm bioluminescence after 4-h; however, this was not statistically significant compared to the control hydrogel. Surprisingly, hydrogels that contained only melittin exhibited no killing compared to biofilms treated with control hydrogels, suggesting that maximum efficacy requires both melittin and tobramycin.

## Discussion

Here, we show that melittin alone and in combination with tobramycin has potent and rapid activity against mature biofilms of both Gram-negative and Gram-positive bacteria. We also show that melittin and tobramycin are effective at micromolar concentrations, potentially lowering the concentration of tobramycin needed for treatment, which could potentially reduce its nephro-ototoxic side effects (Gabriel, 1982).

Because the biofilm biomass causes frustrated phagocytosis and neutrophilic collateral tissue damage (Stoltz et al., 2015), compounds that disrupt biofilms, such as inhaled DNase, and those that reduce the immune response, such as anti-inflammatory drugs, are the cornerstone of CF therapies (Chmiel et al., 2013). Importantly, we found that melittin causes both biofilm dispersal and permeabilization, suggesting that melittin may provide potential anti-inflammatory benefits by dispersing the biofilm and enabling a more effective immune response. It has also been reported that AMPs can disrupt metabolism, cell wall, nucleic acid and protein synthesis (Yeaman and Yount, 2003). These activities may also contribute to the effectiveness of melittin alone and in combination with tobramycin.

Surprisingly, we also found that melittin only enhanced aminoglycosides against PAO1 *P. aeruginosa* growing as biofilms, suggesting a biofilm specific mechanism. However, this was primarily due to the fact that the aminoglycosides themselves were highly effective against planktonic bacteria. Thus, for treatment of a wound, we expect that the combination used at concentrations needed to kill biofilms would also be able to kill planktonic cells. We also show that the combination enhanced killing of the known tobramycin resistant *P. aeruginosa* strain AMT0023_34. This strain over expresses the RND-type MexXY-OpRM efflux pump, rendering it resistant to tobramycin (Mulcahy et al., 2010). Overriding resistance could be very beneficial in the setting of a chronic infection. Interestingly though, the synergy of tobramycin and melittin against biofilms of AMT0023_34 was less pronounced than what we previously observed for tobramycin combined with triclosan (Maiden et al., 2018a), suggesting triclosan is better able to overcome tobramycin resistance caused by efflux pump overexpression.

Importantly, we show melittin combined with tobramycin in a hydrogel is effective at reducing *P. aeruginosa* biofilms *in vivo* using a murine wound model. Antimicrobial hydrogels are a promising emerging biomedical technology for the treatment of microbial infections, especially those associated with wounds (Tse and Engler, 2010; Li et al., 2011; Baysal et al., 2013; Salomé Veiga and Schneider, 2013; Wang et al., 2017). Hydrogels that contained only melittin were ineffective. We speculate that this may be due to protease activity in the wound released by neutrophils (Zhao et al., 2016). Interestingly, aminoglycosides are known to be heparin mimics that can inhibit proteases released by neutrophils and *Bacillus anthracis* (Lee et al., 2004; Craciun et al., 2016). This may explain why the combination is effective, whereas melittin alone is not.

There are numerous studies demonstrating melittin’s anti-bacterial properties against pathogens including *Borrelia burgdorferi*, *S. aureus*, *Escherichia coli*, *K. pneumoniae* and *P. aeruginosa* (Choi et al., 2015; Dosler et al., 2016; Socarras et al., 2017). Melittin also exhibits anti-inflammatory properties in acne vulgaris, atherosclerosis and arthritis in animal models (Lee and Bae, 2016). This study adds to the growing literature on melittin demonstrating that melittin alone and in combination with antibiotics has enhanced activity against biofilms (Dosler and Mataraci, 2013; Dosler and Karaaslan, 2014; Dosler et al., 2016; Choi et al., 2015; Socarras et al., 2017; Akbari et al., 2018).

Antimicrobial peptides are effective and routinely used clinically. For example, colistin or polymyxin E is used for the treatment of Gram-negative bacteria infections in patients with CF (Falagas et al., 2005; Herrmann et al., 2010) and polymyxin B is used in Neosporin^®^ for wounds (Reffuveille et al., 2014; Tran et al., 2016). Taken together, these data demonstrate that alone or in combination with aminoglycosides, melittin could be a new AMP therapy for the treatment of biofilm-associated infections in chronic wounds using a hydrogel. To our knowledge this study provides the first evidence that melittin in combination with tobramycin in a hydrogel is an effective treatment for biofilm infections in an *in vivo* animal model.

## Author Contributions

MM and MZ performed all the experiments, analyzed all the data, and created all the figures. MM and CW wrote and edited the manuscript.

## Conflict of Interest Statement

The authors declare that the research was conducted in the absence of any commercial or financial relationships that could be construed as a potential conflict of interest.
