# Supplementary material for: Hydrogels Embedded With Melittin and Tobramycin Are Effective Against Pseudomonas aeruginosa Biofilms in an Animal Wound Model
Source: Front Microbiol. 2019 Jun 20;10:1348. doi: 10.3389/fmicb.2019.01348 (PMC6598697; doi:10.3389/fmicb.2019.01348)
Supplement: Supplementary file 1 [file Presentation_1.PPTX]

## Slide 1
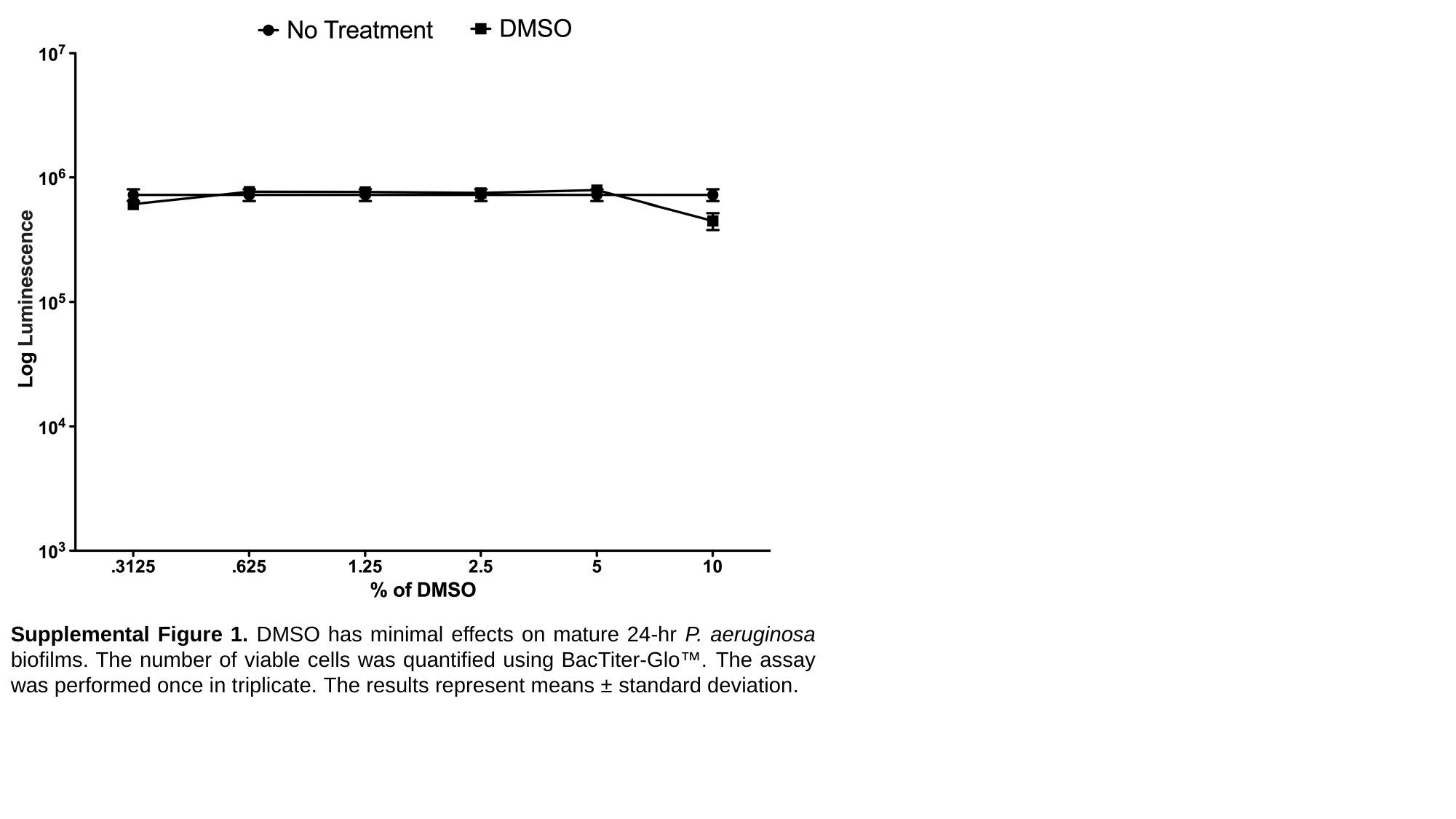

Supplemental Figure 1. DMSO has minimal effects on mature 24-hr P. aeruginosa biofilms. The number of viable cells was quantified using BacTiter-Glo™. The assay was performed once in triplicate. The results represent means ± standard deviation.

## Slide 2
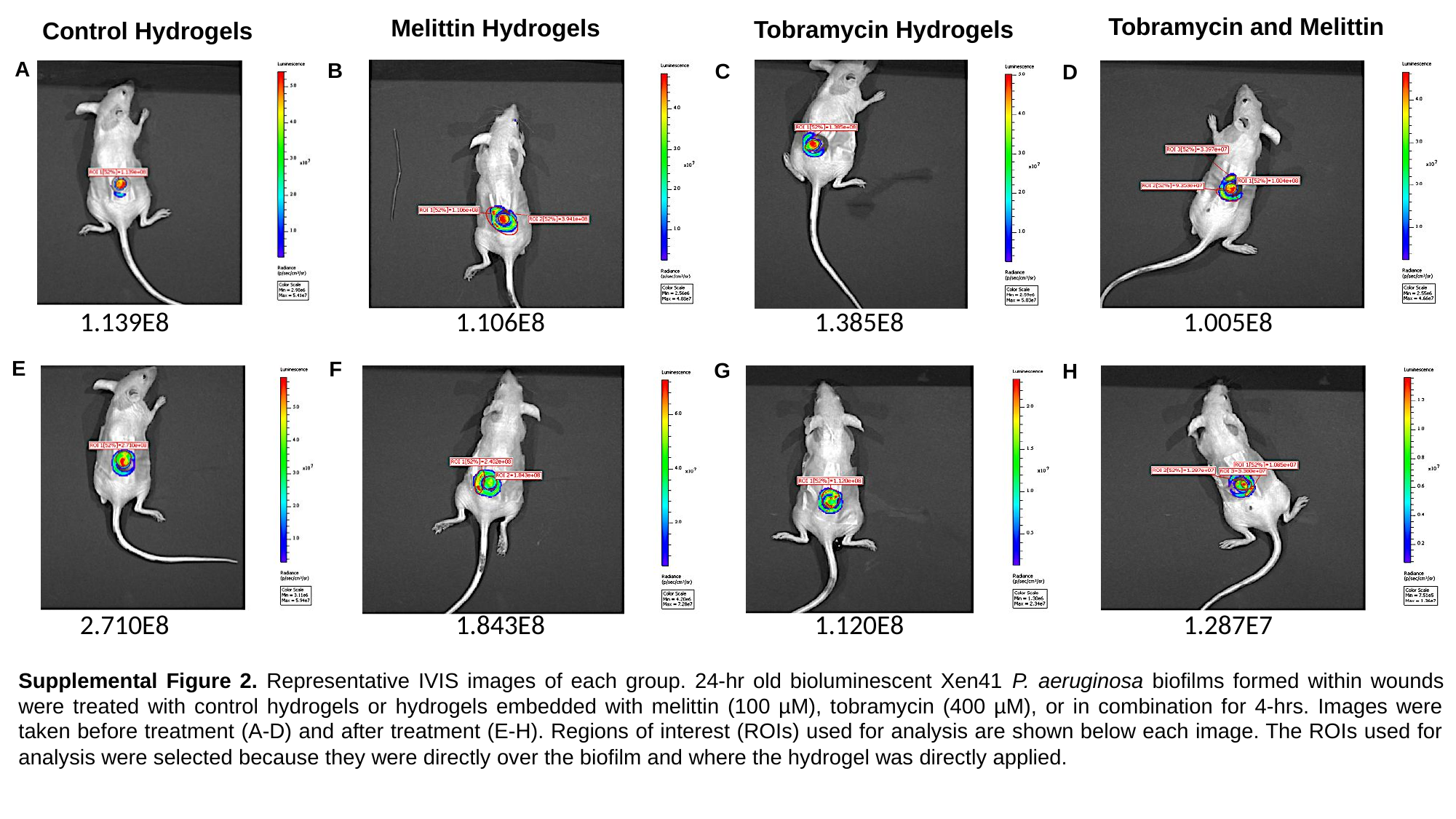

Tobramycin and Melittin
Melittin Hydrogels
Tobramycin Hydrogels
Control Hydrogels
A
B
C
D
1.385E8
1.005E8
1.139E8
1.106E8
E
F
G
H
1.120E8
1.287E7
2.710E8
1.843E8
Supplemental Figure 2. Representative IVIS images of each group. 24-hr old bioluminescent Xen41 P. aeruginosa biofilms formed within wounds were treated with control hydrogels or hydrogels embedded with melittin (100 µM), tobramycin (400 µM), or in combination for 4-hrs. Images were taken before treatment (A-D) and after treatment (E-H). Regions of interest (ROIs) used for analysis are shown below each image. The ROIs used for analysis were selected because they were directly over the biofilm and where the hydrogel was directly applied.

## Slide 3
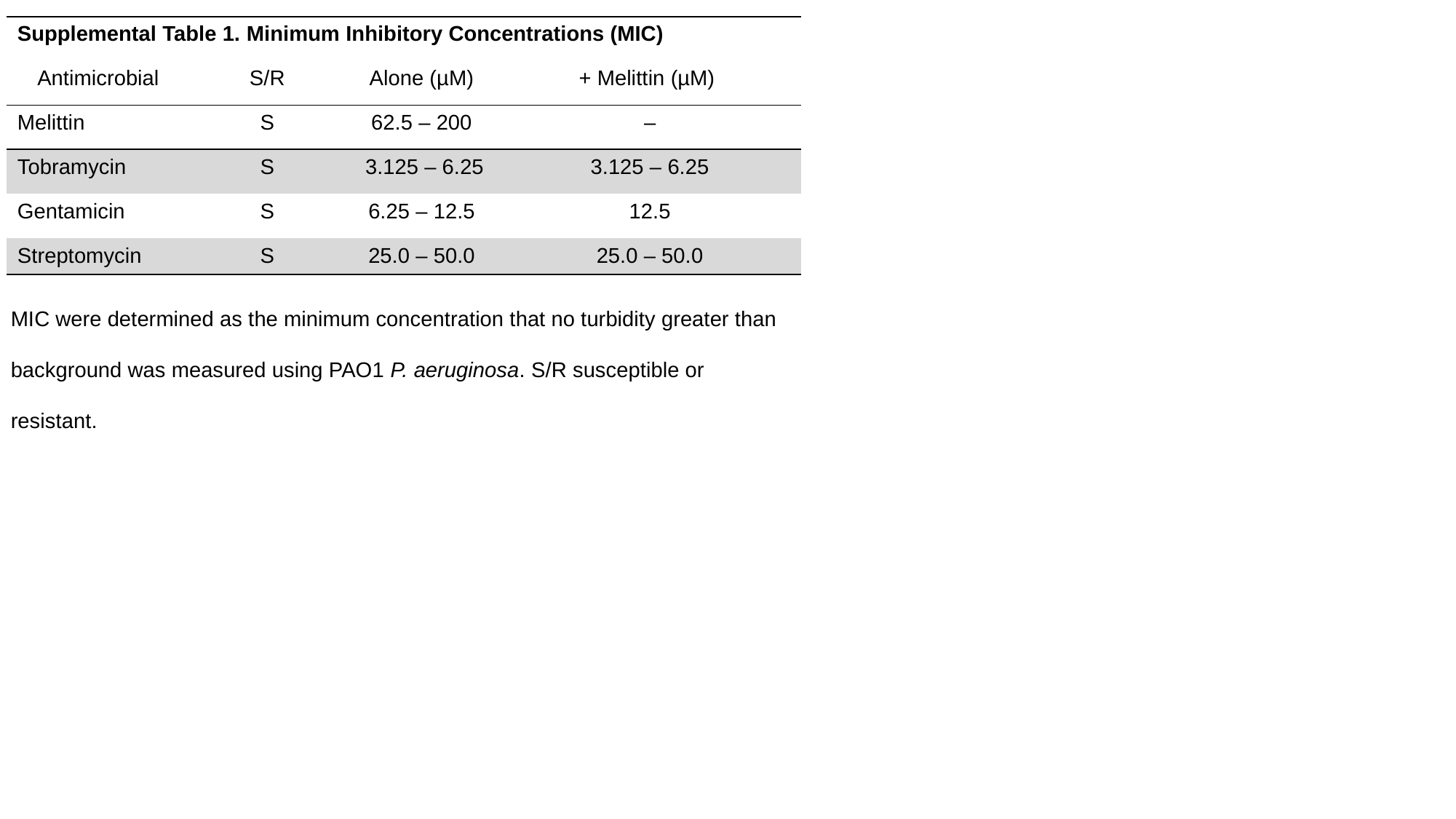

| Supplemental Table 1. Minimum Inhibitory Concentrations (MIC) | | | |
| --- | --- | --- | --- |
| Antimicrobial | S/R | Alone (µM) | + Melittin (µM) |
| Melittin | S | 62.5 – 200 | – |
| Tobramycin | S | 3.125 – 6.25 | 3.125 – 6.25 |
| Gentamicin | S | 6.25 – 12.5 | 12.5 |
| Streptomycin | S | 25.0 – 50.0 | 25.0 – 50.0 |
MIC were determined as the minimum concentration that no turbidity greater than background was measured using PAO1 P. aeruginosa. S/R susceptible or resistant.

## Slide 4
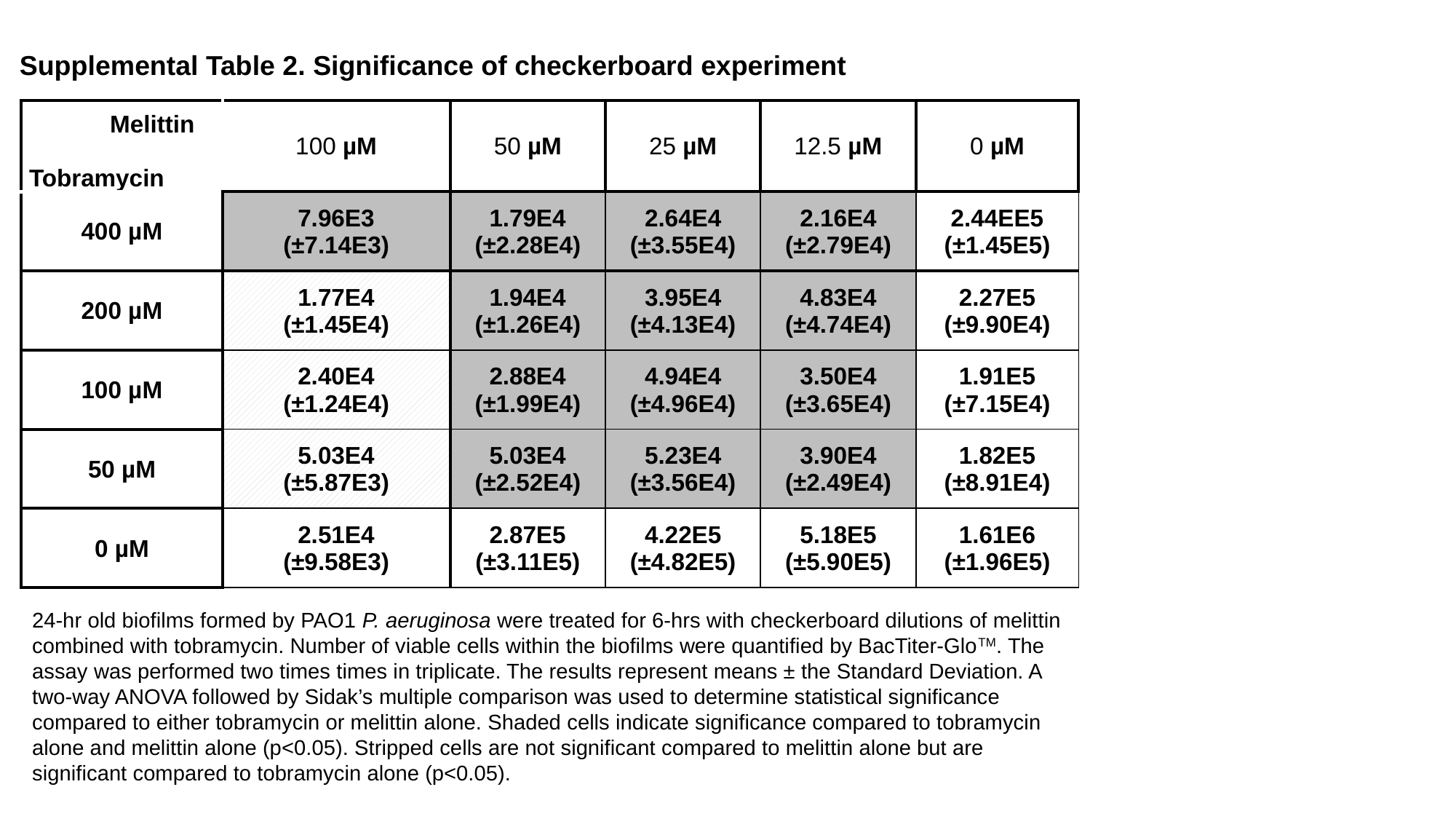

Supplemental Table 2. Significance of checkerboard experiment
| Melittin Tobramycin | 100 µM | 50 µM | 25 µM | 12.5 µM | 0 µM |
| --- | --- | --- | --- | --- | --- |
| 400 µM | 7.96E3 (±7.14E3) | 1.79E4 (±2.28E4) | 2.64E4 (±3.55E4) | 2.16E4 (±2.79E4) | 2.44EE5 (±1.45E5) |
| 200 µM | 1.77E4 (±1.45E4) | 1.94E4 (±1.26E4) | 3.95E4 (±4.13E4) | 4.83E4 (±4.74E4) | 2.27E5 (±9.90E4) |
| 100 µM | 2.40E4 (±1.24E4) | 2.88E4 (±1.99E4) | 4.94E4 (±4.96E4) | 3.50E4 (±3.65E4) | 1.91E5 (±7.15E4) |
| 50 µM | 5.03E4 (±5.87E3) | 5.03E4 (±2.52E4) | 5.23E4 (±3.56E4) | 3.90E4 (±2.49E4) | 1.82E5 (±8.91E4) |
| 0 µM | 2.51E4 (±9.58E3) | 2.87E5 (±3.11E5) | 4.22E5 (±4.82E5) | 5.18E5 (±5.90E5) | 1.61E6 (±1.96E5) |
24-hr old biofilms formed by PAO1 P. aeruginosa were treated for 6-hrs with checkerboard dilutions of melittin combined with tobramycin. Number of viable cells within the biofilms were quantified by BacTiter-GloTM. The assay was performed two times times in triplicate. The results represent means ± the Standard Deviation. A two-way ANOVA followed by Sidak’s multiple comparison was used to determine statistical significance compared to either tobramycin or melittin alone. Shaded cells indicate significance compared to tobramycin alone and melittin alone (p<0.05). Stripped cells are not significant compared to melittin alone but are significant compared to tobramycin alone (p<0.05).
